# Primary Penile Squamous Cell Cancer-Related Malignant Priapism in a Cystectomized Patient: A Case Report

**DOI:** 10.7759/cureus.31875

**Published:** 2022-11-24

**Authors:** Selman Unal, Amjad S Alijla, Berrak G Ocal, Emrah Okulu, Onder Kayigil

**Affiliations:** 1 Urology, Ankara Yildirim Beyazit University Faculty of Medicine, Ankara, TUR; 2 Urology, Johns Hopkins University School of Medicine, Baltimore, USA; 3 Urology, Artvin Public Hospital, Artvin, TUR; 4 Pathology, Ankara Yildirim Beyazit University Faculty of Medicine, Ankara, TUR

**Keywords:** squamous cell carcinoma, bladder neoplasm, priapism, carcinoma of the penis, penile mass

## Abstract

Priapism is one of the most common urologic emergencies and is characterized by a prolonged and painful erectile state unrelated to sexual stimulation or sexual desire. Neoplasm-associated priapism is a rare condition and is usually caused by corporeal metastases of other pelvic area malignancies. Primary penile malignancy-related malignant priapism is extremely rare. In this reported case, an 82-year-old male presented with priapism. The penile doppler ultrasound and pelvic magnetic resonance imaging were compatible with ischemic priapism and corporal mass. Subsequently, the patient underwent total penectomy and bilateral superficial inguinal lymphadenectomy. The pathology report was consistent with primary penile squamous cell cancer (SCC), so the patient underwent adjuvant radiotherapy. However, he developed multiple metastases and could survive for about six months. The patient had undergone radical cystectomy (RC) and urethrectomy 19 and 2 years ago due to urothelial carcinoma, respectively. To the best of our knowledge, this is the second case of malignant priapism due to primary penile SCC and represents one of the longest urethral recurrence periods after RC. When a patient presents with malignant priapism, primary penile malignancies should be considered in differential diagnosis, even if the patient has a history of pelvic area malignancies.

## Introduction

Priapism is prolonged (more than four hours) and unintended erection without sexual stimulation or the emergence of sexual desire [1]. It could be secondary to hematological diseases such as sickle cell anemia, infections, metabolic diseases, drugs, and neoplasms [2,3]. Priapism due to neoplasms is called malignant priapism, which is a rare condition that tends to occur with corporal metastasis of other pelvic area malignancies [4]. Priapism due to primary penile malignancies is extremely rare [5].

In this report, we present the case of a patient who underwent total penectomy and bilateral inguinal lymphadenectomy due to malignant priapism related to primary penile squamous cell cancer (SCC), as well as radical cystectomy (RC) and urethrectomy 19 and 2 years ago due to urothelial carcinoma (UC), respectively.

## Case presentation

**Figure 1:**
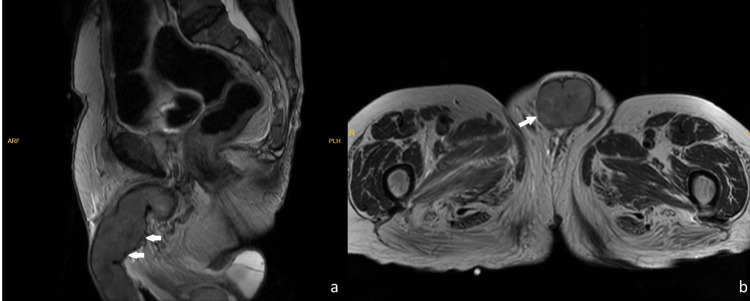
Pelvic magnetic resonance imaging of the patient. Penile squamous cell carcinoma invading the corpus cavernosum (the white arrows indicate the mass) on magnetic resonance imaging (MRI) (1.5-Tesla MRI (Signa, GE Medical Systems, USA)). a: Sagittal plane, b: Transverse plane.

An 82-year-old male patient presented to the outpatient clinic with an unintended and painful penile erection for 48 hours duration. The patient’s clinical history indicated that he had undergone RC and orthotopic neobladder 19 years ago due to muscle-invasive UC of the bladder. No tumor was observed in the final cystectomy pathology (T0); therefore, no adjuvant therapy was given. He underwent total urethrectomy, neobladder resection, and ileal conduit 17 years after the RC operation due to UC recurrence on the mid-urethra. The pathology report of the urethra was consistent with high-grade urothelial carcinoma with subepithelial invasion (pT1 high-grade). Adjuvant cisplatin-based chemotherapy was administered. On the examination of the patient, a cold and erect penis with a 1 cm diameter ulcerated lesion on the glans penis was observed. Bedside corporal irrigation and aspiration were performed, and a cavernosal blood gas sample was sent for differential diagnosis. Hypoxia, hypercarbia, and acidosis were observed on the cavernosal blood gas examination (pH:7.01, pO2: 27 mmHg, pCO2: 78 mmHg), which was compatible with ischemia. The hemogram was normal. Detumescence could not be achieved, and for the evaluation of additional pathologies, the patient underwent the emergent penile duplex Doppler ultrasound, which showed decreased blood flow and a mass on the proximal part of the penis. Subsequently, pelvic magnetic resonance imaging was performed, and a mass was observed, which was fully destructive to the cavernosal bodies and had irregularly circumscribed borders and necrotic areas; the border with the pubis was indistinct (Figure 1).

**Figure 2:**
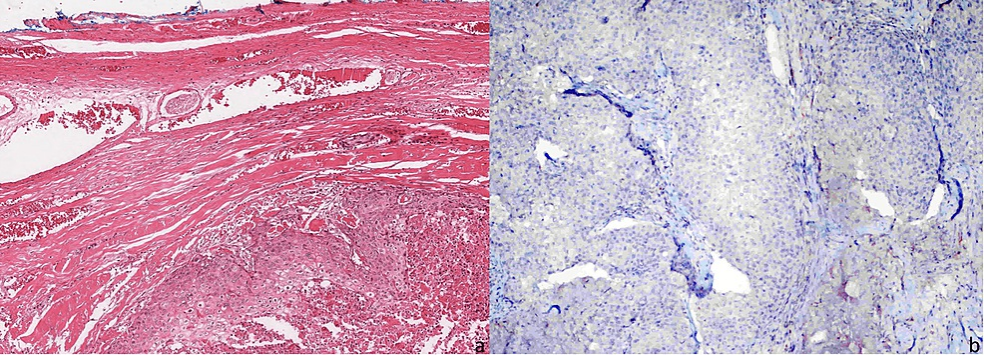
Microscopic examination of the penectomy specimen. a: Pure squamous cell carcinoma with extensive necrosis is filling the penile shaft. Urothelial carcinoma morphology is absent, and no relation with skin/surface is found (HEx10); b: Immunohistochemically preparation obtained from a penectomy specimen that was evaluated for p16 and p16 is absent (IHCx20).

Later, the patient underwent total penectomy and bilateral inguinal lymphadenectomy. The report from the penectomy specimen suggested that the tumor was compatible with moderately differentiated SCC. Extensive sampling of the tumor did not reveal any UC in situ or SCC in situ. The bilateral corpus cavernosum was fully infiltrated by the tumor, with SCC morphology starting from the surgical margin and damaging all structures of the penile body (Figure 2a). For a possible contribution to differential diagnosis, p16 immunohistochemistry was performed, but the tumor was negative. Since p16 is used as a surrogate marker for human papillomavirus-(HPV) driven cancers, and in this case, its positivity would have supported primary SCC driven by HPV. However, since it was negative, it did not help the pathologist make a definitive diagnosis (Figure 2b). No sign of metastasis was observed in the lymph nodes (pT3N0). The tumor was positive on the proximal surgical margin. Subsequently, the patient underwent adjuvant pelvic radiotherapy; however, because his general condition deteriorated, he could not continue the radiation therapy and receive chemotherapy; subsequently, he developed multiple metastases and could survive for only about six months.

## Discussion

Malignant priapism is a rare clinical condition caused by primary or secondary malignancies of the penis. It was first described by Peacock in 1938 as a persistent and painful erection resulting from an invasion of the cavernosal sinuses and efferent veins [4]. Malignant priapism is caused by secondary penile tumors rather than primary ones. The most common origins of corporal metastases are the bladder, the prostate, and recto-sigmoid tumors, in that order [6]. The size, location, presence of priapism, and prognosis of the underlying tumor affect the therapy of penile metastases [7]. Local excision, partial or complete penectomy, radiation, chemotherapy, and a conservative approach are some of the therapy options [7,8].

Cases of priapism due to primary penile malignancies are extremely rare. Priapism due to primary penile malignancies has been reported in six cases in the literature [9-11]. While four cases had underlying primary penile lymphoma, one case had penile hemangioendothelioma, and only one case had primary penile SCC. In the case of primary penile SCC, a 37-year-old male patient presented with priapism, and a penile biopsy revealed a poorly differentiated SCC. Since the general condition of the patient was poor, external beam radiotherapy was applied; however, the patient’s condition worsened in the next two months, and he succumbed to his disease [10]. There are no established protocols for treating malignant priapism caused by penile squamous cell carcinoma. Since malignant priapism due to primary penile malignancies is a locoregional illness as opposed to a secondary one that induces priapism, multimodality therapy can be curative. In the case presented in this report, we performed total penectomy as well as bilateral inguinal lymphadenectomy on this 80-year-old patient who had severe pain due to malignant ischemic priapism and whose penis had no urinary function due to previous RC and urethrectomy operations, followed by pelvic radiotherapy. However, this treatment was not sufficient to provide cancer control, and the patient developed multi-metastases.

## Conclusions

In conclusion, we reported a patient who had primary penile SCC-related malignant priapism and a history of bladder and urethral cancer. Despite receiving aggressive local treatment, the patient could survive for only six months. Although it is the most common primary malignancy of the penis, to the best of our knowledge, priapism due to primary penile SCC has been reported only once in the literature. Although the metastatic disease is typically thought to be the cause, when a patient with a history of pelvic malignancy presents with malignant priapism, it should be kept in mind that primary malignancies of the penis may also be the etiology. As the data on primary penile cancer-related malignant priapism patients increase, a therapeutic guideline could be created to treat this condition.
